# Impact of pulsed xenon ultraviolet disinfection on surface contamination in a hospital facility’s expressed human milk feed preparation area

**DOI:** 10.1186/s12879-018-2997-9

**Published:** 2018-02-23

**Authors:** Ricky Dippenaar, Johan Smith

**Affiliations:** 1 Department of Neonatology, Netcare Blaauwberg & N1 City Hospital, Waterville Crescent, Sunningdale, Western Cape 7441 South Africa; 2 Department of Paediatrics & Child Health, Stellenbosch University & Tygerberg Children’s Hospital, Tygerberg, Western Cape 7505 South Africa

**Keywords:** Feed preparation areas, Hospital infection, Bioburden, Non touch disinfection, Pulsed xenon ultraviolet

## Abstract

**Background:**

Expressed human milk (EHM) feed preparation areas represent a potential source of unintentional nosocomial infection. Daily disinfection of environmental surfaces remains an essential intervention to mitigate nosocomial infections. The inefficiency of conventional cleaning and disinfection contributes to an increased risk for the acquisition of multi-drug resistant pathogens. “Non touch” technologies such as the pulsed xenon ultraviolet (PX-UVD) light device have documented sustained reduction in surface bacterial colonization and reduced cross contamination.

**Methods:**

The impact of a PX-UVD on surface colony forming units per square centimeter (cfu/cm^2^) in feed preparation areas was evaluated following its implementation as standard care. A quasi-experimental study was performed documenting bacterial colonization from 6 high risk feed preparation areas in a community care hospital in South Africa. Pre and post conventional cleaning neutralizing rinse swabs were collected fortnightly over a 16 week control period prior to the introduction of the PX-UVD and compared to a matching set of samples for the PX-UVD period.

**Results:**

A 90% reduction in total surface bioburden was noted from the control period (544 cfu/cm^2^) compared to the corresponding PX-UVD period (50 cfu/cm^2^). Sub -analysis of both the Pre-clean Control: Pre-clean PX-UVD counts as well as the Post-clean Control: Post-clean PX-UVD counts noted significant improvements (*p* < 0.001). A statistically significant improvement was noted between pre-and post-cleaning total surface bioburden following exposure to the PX-UVD (*p* = 0.0004). The introduction of the PX-UVD was associated with a sustained reduction in the pre clean bioburden counts with a risk trend (per week) 0.19, (95% CI [0.056, 0.67], *p* = 0.01).

**Discussion:**

The use of a PX-UVD as adjunct to standard cleaning protocols was associated with a significant decrease in surface bioburden. The study demonstrated the inefficiency of conventional cleaning. Persistence of potentially pathological species in both periods highlights current health sector challenges.

## Background

Expressed human milk (EHM) feed preparation areas remain an integral aspect of neonatal intensive care as well as pediatric critical care units, and are common place in any hospital setting. These areas also represent a key source of infection and contamination resulting in unintentional nosocomial infections [1, 2].

The South African National Department of Health (DOH) has strict procedural requirements for any designated feed preparation area including the use of sterile gowns and gloves during the preparation of feeds by trained individuals in designated well marked areas. The Netcare private hospital group has additional standard operational procedures (SOP) for the daily disinfection of feed preparation areas as well as the safe preparation, storage and handling of expressed human milk. Although daily disinfection of environmental surfaces remains an essential intervention to mitigate nosocomial infections [3], the inefficiency of recognized cleaning and disinfection practices remains concerning [4]. Mitchell et al., 2015, found that failure to adequately disinfect high risk areas contributes to an increased risk for the acquisition of multi-drug resistant pathogens [5]. The inclusion of “non touch” room disinfection technology represents a proven adjunct to any facility’s disinfection SOP aimed at addressing potential shortcomings [6].

The pulsed xenon ultraviolet (PX-UVD) light device is a “non touch” ultraviolet C (UV-C) emitting technology designed for the hospital setting. Each pulse from the non-mercury Xenon flash lamp releases approximately 505 J of energy into high intensity broad-spectrum UV light, with a narrow band concentration within the UV-C spectrum [7]. The germicidal effects of UV-C irradiation (200–300 nm) results in cellular damage by photohydration, photosplitting, photodimerization and photo crosslinking, thereby inhibiting cellular replication [8]. Implementation of this “non touch” technology in various hospitals has documented a sustained reduction in surface bacterial colonization [9], reduced cross contamination [10] and reduced spread of multi drug resistant bacterial infections in settings other than a feed preparation area [11, 12].

## Method

### Aim

The aim of this study was to evaluate the effect of a pulsed-xenon ultraviolet portable device (PX-UVD) as compared to standard care on surface colony forming units per square centimeter (cfu/cm^2^) within neonatal and pediatric EHM feed preparation areas at Netcare Blaauwberg hospital.

### Study setting

Netcare Blaauwberg private hospital is a 140 bed acute care community hospital in the Western Cape of South Africa, with a 12 bed neonatal intensive care unit (NICU), a 16 bed pediatric ward and a 16 bed maternity ward.

The NICU, maternity and pediatric wards actively participate in the baby friendly initiative promoting human milk exclusivity. The NICU utilizes a multi counter dedicated expressed human milk (EHM) feed preparation area for the processing of stored fresh and frozen EHM. The maternity and pediatric wards have a dedicated single counter feed preparation area. Reconstitution of dry milk formulae only occurs within the pediatric and maternity wards on strict prescription of the attending pediatricians.

### Design

A quasi-experimental study was conducted from June 2015 until February 2016. The study was approved as a nonhuman-subject, quality-improvement study by the Netcare research operations committee and the University of Stellenbosch ethics committee.

### Sample

Environmental surface bioburden was evaluated by collecting pre – and post cleaning surveillance swabs from 6 surfaces in 3 feed preparation areas using pre-immersed neutralizing rinse swabs (NRSII**™** Transwab ®). The six high risk areas identified included:, the NICU prewash EHM bottle area, the NICU post-wash EHM bottle area, the NICU EHM preparation area, the NICU fridge door handle and the single counter surface within the feed preparation areas of both the pediatric and maternity wards.

Pre cleaning swabs were collected fortnightly at 7 am for the duration of the study. The study coordinator determined the day of the week for sampling using a simple sealed envelope randomization system which was then relayed to the head of infection control. The head of infection control performed all sampling for the study duration. All sampling was standardised to a single predetermined 10 cm (cm) × 10 cm area for each surface as per the recommendation of the resident clinical microbiologist.

Following pre clean sampling, the area was cleaned as per the facility’s SOP. The facility’s SOP for daily terminal cleaning of working surfaces in the feed preparation areas involves initial cleaning with soap and water using commercially available disposable cloths, followed by disinfection with a suspension of Troclosene Sodium (NaDCC) at 500 ppm (ppm). Cleaning of the fridge door and handle is a specifically allocated area and includes the aforementioned protocol in addition to weekly cleaning of the inside of the fridge and monthly defrosting. One designated trained multi-shift cleaning team is allocated to this duty on a continuous basis. The area is then allowed to air dry for 1 h after which post cleaning swabs were taken from the same allocated areas.

Cleaning staff and nursing staff were blinded to the details of the study as well as to the timing of the swabs, allocated areas and frequency of sampling. The facility’s head of infection control and resident microbiologist remained blinded to the sample results for the duration of the study.

### Measurement

A total of 108 CONTROL samples were collected fortnightly over a 16 week period prior to the implementation of the PX-UVD on week 17 of the study. The introduction of the PX-UVD to the standard cleaning protocol involved the daily cleaning of the allocated feed preparation areas as per facility’s SOP including an air dry period for 1 h. Thereafter the PX-UVD was placed on either side of each of the 3 feed preparation areas for a 5-min treatment cycle, as per manufacturers recommendations. Post cleaning swabs were taken immediately after exposure to the PX-UVD. A matching 108 PX-UVD samples was collected over the ensuing 16 weeks.

### Environmental testing procedure

The pre-immersed neutralizing rinse swabs (NRSII™ Transwab ®) were immediately collected and transported by Pathcare laboratory services in a temperature regulated environment for processing at their off-site facility. Each swab container underwent mixing by vortexing 1 ml of neutralizing rinse solution which was then placed on a total viable count (TVC) Petrifilm (3 M Rehydratable film method) agar. Petrifilm agars were then incubated at 35° ± 2 °C for 48 ± 3 h. The total viable count was then quantified into number of colony forming units per square centimeter (cfu/cm^2^). The colonies cultured, included both natural environmental contaminant species as well as potentially pathogenic species, were then transferred to agar plates for further organism identification.

### Device

A single PX-UVD (Xenex Disinfection Services, San Antonio, Texas**)** was received on loan from Kiara Healthcare for the duration of the 4-month study period. Floor plans, counter heights, and room dimensions were relayed to the manufacturer. The optimal efficacy for the device was mathematically modelled based on spectrometer data and the location and size of the target areas. The resulting recommendation of two treatment cycles of 5-min per side of each allocated feed preparation area was determined to ensure maximum counter exposure with no shadow areas.

### Data analysis

Total surface bioburden was calculated as the sum of the viable colony count (cfu/cm^2^) of the 6 counter surfaces in the pre and post cleaning phases. Statistical analyses was performed using the NCSS statistical analysis package (NCSS 11 Statistical Software (2016). NCSS, LLC. Kaysville, Utah, USA.)

Numerical data was log transformed to achieve normality. A multi-variance ANOVA analysis was applied to the log sample data to determine statistical relevance and trend analysis. The log data was back transformed and the observed geometric mean differences represented as risk ratios.

## Results

A 90% reduction in total surface bioburden was noted from the control period (544 cfu/cm^2^) compared to the corresponding PX-UVD period (50 cfu/cm^2^). Pre cleaning surface bioburden significantly improved from 244 cfu/cm^2^ in the CONTROL period to 44 cfu/cm^2^ in the PX-UVD period with a geometric mean risk ratio 0.11, (95% CI [0.04, 0.29], *p* < 0.001). Similarly, the post cleaning surface bioburden significantly improved from 300 cfu/cm^2^ in the CONTROL period to 6 cfu/cm^2^ in the PX-UVD period with a geometric mean risk ratio 0.04*,* (95% CI [0.02, 0.09], *p* < 0.001). Individual counter surface data during the CONTROL period noted higher average surface bioburden within areas of the NICU, most noteworthy the post-wash EHM bottle area and the EHM preparation area recorded higher surface bioburden counts post conventional cleaning. (Table 1) The highest average surface bioburden count was consistently measured on the Fridge door handle. Individual counter surface data for the matching PX-UVD period demonstrated a sustained improvement post cleaning as well as significantly reduced average surface bioburden counts across all surfaces measured (Table 1).

**Table 1:** Geometric mean (GM) of colony counts per sample area

Area	Control						PX-UVD					
	PreClean GM^a^	PostClean GM^a^	∆ GM^b^	Risk Ratio	*95% CI* ^c^	*p*-value	PreClean GM^a^	PostClean GM^a^	∆ GM^b^	Risk Ratio	*95% CI* ^c^	*p*-value
PreWashBottle	0.37	0.04	0.33	0.09	0.01, 0.61	0.0143	0.05	0.02	0.03	0.33	0.05, 2.13	0.2395
PostWashBottle	0.58	1.68	1.10	2.89	0.44, 18.85	0.2646	0.07	0.01	0.07	0.10	0.02, 0.68	0.0188
EHMprep	0.65	0.78	0.13	1.19	0.18, 7.77	0.8533	0.09	0.02	0.07	0.23	0.03, 1.47	0.1187
FridgeDoorHandle	1.23	1.15	0.08	0.93	0.14, 6.09	0.9432	0.14	0.04	0.10	0.28	0.04, 1.83	0.1820
Maternity	0.81	0.18	0.63	0.22	0.03, 1.44	0.1138	0.16	0.01	0.15	0.03	0.00, 0.19	0.0003
Pediatric	0.45	0.12	0.33	0.27	0.04, 1.78	0.1727	0.02	0.01	0.01	0.69	0.11, 4.49	0.6948

^a^geometric mean (GM), ^b^difference in geometric mean (∆ GM), ^c^ Confidence Interval (CI)

**Fig. 1:**
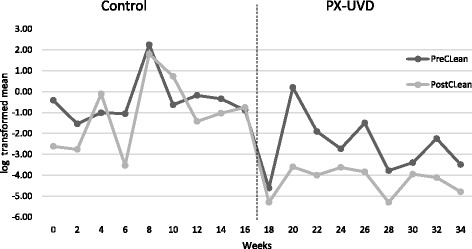
Log transformed mean bioburden over weeks

The graphical representation of the CONTROL period (Fig. 1) demonstrates an inconsistent response to conventional cleaning including a worsening of the post cleaning total surface bioburden in weeks 4 and 10. The introduction of the PX-UVD at 17 weeks was initially associated with a dramatic reduction in both the pre and post cleaning total surface bioburden, followed by a sustained reduction in the pre clean surface bioburden counts with a risk trend (per week) 0.19, (95% CI [0.056, 0.67], *p* = 0.01). (Figure 1) Furthermore, in contrast to the CONTROL period (geometric mean risk ratio 0.08, (95% CI [0.24, 1.10], *p* = 0.08)) a statistically significant improvement was demonstrated between the pre cleaning total surface bioburden and the post cleaning total surface bioburden following exposure to the PX-UVD (geometric mean risk ratio 0.19, (95% CI [0.09, 0.40], *p* = 0.00004)), including complete eradication of detectable bacteria in weeks 18 and 28.

Twenty three pathological organisms were identified during the control period in comparison to the 5 identified during the PX-UVD period (Table 2).

**Table 2:** Organisms identified

Control	PX-UVD
7 Acinetobacter baumannii 4 *Enterobacter cloacae* 4 Stenotrophomonas maltophilia 2 Aeromonas hydrophilia 1 Enterococccus casseliflavus 1 Falvimonas oryzihabitans 1 Klebsiella pneumonia ozaenia 1 *Klebsiella pneumoniae* pneumoniae 1 Serratia marcescens 1 Serratia liquifaciens	3 Acinetobacter baumannii 1 Acinetobacter ursingii 1 Klebsiella teringa

## Discussion

Expressed human milk, particularly within the confines of the high-risk environment of the neonatal ICU, represents a critical irreplaceable aspect of the care for these highly vulnerable and immunocompromised infants. Ensuring a sterile dedicated environment for the processing and handling of EHM cannot be overemphasize. Despite our compliance with the South African Department of Health’s and facility’s recommendations for surface disinfection, this study highlighted the inefficiency of conventional cleaning on both natural environmental contaminants and potentially pathogenic species. The significantly higher total surface bioburden counts and increased post clean total surface bioburden counts during the control period invariably contributed to the diversity of potentially pathogenic isolates identified during this period.

The introduction of a “no-touch” PX-UVD as an adjunct to the facility’s conventional cleaning SOP was associated initially with a dramatic reduction in both the pre and post clean total surface bioburden. Subsequently, a sustained reduction in the pre clean surface bioburden counts together with a stabilization and consistent improvement between the pre- and post cleaning surface bioburden, culminated in a statistically significant reduction in pre and post cleaning total surface bioburden for the PX-UVD period. The susceptibility of both environmental contaminants and potentially pathogenic organisms to the germicidal effects of UV-C exposure remains cautiously reassuring of the potential long term sustained effects of PX-UVD.

The relative dominance of potentially pathogenic gram-negative isolates, as opposed to gram-positive organisms such as Clostridia difficile and Methicillin-resistant *Staphylococcus aureus* with documented sensitivity to UV-C [4–7], was presumably the effect of study design, focussing on the neonatal, maternity and paediatric wards with a relatively low facility prevalence. The persistence of the Acinetobacter species in both the CONTROL and PX-UVD periods highlights the challenges the health sector is facing despite the inclusion of newer disinfection solutions and technologies; further hampered by multiple reports of resistance of this genus to conventional disinfection solutions [13] and documented varying susceptibility of microorganisms to ultraviolet disinfection [14].

### Limitations

The limitations of this study include the relatively small study numbers, limited study duration and the lack of variability of performing a single institution study. We did not evaluate the potential long term cumulative suppressive effects following the introduction of the PX-UVD as well as its impact on both environmental and potentially pathogenic organisms; nor the potential impact of a lower surface bioburden and its effect on nosocomial infection rates.

Despite these limitations, as a quality improvement study, several strategies have been strongly recommended and subsequently implemented. Expressed human milk feed preparation areas have been deemed high priority areas. The facility’s SOP has been amended to include the conversion to a commercially available quaternary ammonium disinfection solution to negate the potential risk of over-dilution of NaDCC, nonwoven microfiber spunlace cloths have replaced the commercially available disposable cloths for disinfection and the specialized cleaning teams have been re-educated emphasizing on key impact measures such as disinfectant contact time. In addition, a quality assurance monitoring system using adenosine triphosphate (ATP) bioluminescence was introduced to evaluate cleaning practices within the EHM feed preparation area, providing feedback to the specialized cleaning teams. The acquisition and permeant inclusion of a PX-UVD as standard care has been strongly recommended.

## Conclusion

The use of a PX-UVD as an adjunct to the facility’s standard cleaning protocols within the EHM feed preparation areas was associated with a significant decrease in surface bioburden. Future long term studies are envisioned to evaluated the relationship of a reduced surface bioburden and its impact on nosocomial infection, particularly within neonatal ICU.

## Abbreviations

cfu/cm^2^: Colony forming units per square centimeter
cm: Centimeter
DOH: South African National Department of Health
EHM: Expressed human milk
NaDCC: Troclosene Sodium
NICU: Neonatal intensive care unit
ppm: Parts per million
PX-UVD: Pulsed xenon ultraviolet light device
SOP: Standard operational procedures
UV-C: Ultraviolet C
